# EHMTI-0315. AMG 334, the first potent and selective human monoclonal antibody antagonist against the CGRP receptor

**DOI:** 10.1186/1129-2377-15-S1-G43

**Published:** 2014-09-18

**Authors:** C Xu, L Shi, S Rao, C King, H Sun, D Zhu, S Lehto, K Wild, D Immke

**Affiliations:** 1 Neuroscience, Amgen Inc., Thousand Oaks, USA; 2 Therapeutic Discovery, Amgen Inc., Thousand Oaks, USA

## Background

Clinical studies with multiple CGRP receptor antagonists reported that CGRP receptor antagonism is effective in acute migraine reversal. Using XenoMouse® Technology, we successfully generated a group of human monoclonal antibodies (mAb) that specifically target the human CGRP receptor.

## Aim

to characterize the pharmacological properties of AMG 334, one of the mAbs currently in clinical development for migraine prevention

## Methods

We used [125I]-CGRP and [125I]-AMG 334 binding assays to measure AMG 334/CGRP-receptor interactions, cell-based cAMP assays to study functional activity and selectivity of AMG 334, and a laser Doppler model in cynomolgus (cyno) macaque to assess the pharmacodynamic effects of blocking capsaicin-induced increases in dermal blood flow with AMG 334.

## Results

AMG 334 is a potent inhibitor of [125I]-CGRP binding to the human CGRP receptor with a Ki of 0.02 nM. It exhibited full inhibition of CGRP-stimulated cAMP production with an IC50 of 2.3 nM in cell-based functional assays. Potency of AMG 334 at the cyno CGRP receptor is similar to that at the human receptor, but with significant reduced potency at dog, rabbit and rat receptors. AMG 334 also demonstrates > 5000-fold selectivity over other closely related receptors in the family. The receptor kinetics studies using [125I]-AMG 334 reveals a dissociation t1/2 off of 67 min.

In the cyno study, AMG 334 produces a significant and sustained inhibitory effect on capsaicin-induced increase in dermal blood flow.

## Conclusion

AMG 334 is a potent and selective antibody against the human CGRP receptor with potential for migraine prevention.

